# Brain-wide reconfiguration of burst firing by psilocybin reveals 5-HT2A-dependent circuit dynamics

**DOI:** 10.64898/2026.08.14.744865

**Published:** 2026-09-07

**Authors:** D. Momi, Y. Nahas, D. Wyrick, L. C. Marks, L. D. Claar, R. De Filippo, P. Seyfourian, D. A. Pizzagalli, M. Buice, T. Ott, C. Koch, I. Rembado

## Abstract

Psilocybin produces rapid and lasting therapeutic effects, yet how 5-HT2A receptor activation reshapes brain-wide circuit dynamics during acute drug administration remains poorly understood. Using simultaneous multi-region Neuropixels recordings of 46,360 single units from 35 mice, together with scalp electroencephalography (EEG), pupillometry, and locomotion monitoring, we provide a brain-wide, single-unit and field-potential characterization of psilocybin’s acute effects, with pharmacological dissection using the 5-HT2A antagonist ketanserin. Psilocybin selectively reconfigured burst coding, rather than mean firing rate, across cortical, thalamic, and hippocampal circuits: burst firing decreased in hippocampal CA1–CA3 and was bidirectionally modulated in the thalamus, with the reticular nucleus bursting more and first-order geniculate nuclei bursting less. Critically, most of these burst effects were abolished by ketanserin, consistent with at least partial 5-HT2A receptor dependence. These data suggest that the psychedelic state is not simply a matter of how much neurons fire, but of how they fire, pointing to a region-specific, 5-HT2A-associated reconfiguration of burst coding that may underlie the acute phenomenology of the psilocybin experience.

## Full Text Availability

The license terms selected by the author(s) for this preprint version do not permit archiving in PMC. The full text is available from the preprint server.

